# Obesity does not aggravate vitrification injury in mouse embryos: a prospective study

**DOI:** 10.1186/1477-7827-10-68

**Published:** 2012-08-31

**Authors:** Wenhong Ma, Xing Yang, Xiaoyan Liang

**Affiliations:** 1 Center for Reproductive Medicine, Sixth Affiliated Hospital of Sun Yat-sen University, 17th Shou-gou-ling Road, Guangzhou, 510655, People’s Republic of China

**Keywords:** Maternal, Obesity, Embryo, Blastocyst, Vitrification

## Abstract

**Background:**

Obesity is associated with poor reproductive outcomes, but few reports have examined thawed embryo transfer in obese women. Many studies have shown that increased lipid accumulation aggravates vitrification injury in porcine and bovine embryos, but oocytes of these species have high lipid contents (63 ng and 161 ng, respectively). Almost nothing is known about lipids in human oocytes except that these cells are anecdotally known to be relatively lipid poor. In this regard, human oocytes are considered to be similar to those of the mouse, which contain approximately 4 ng total lipids/oocyte. To date, no available data show the impact of obesity on vitrification in mouse embryos. The aim of this study was to establish a murine model of maternal diet-induced obesity and to characterize the effect of obesity on vitrification by investigating the survival rate and embryo developmental competence after thawing.

**Methods:**

Prospective comparisons were performed between six–eight-cell embryos from obese and normal-weight mice and between fresh and vitrified embryos. Female C57BL/6 mice were fed standard rodent chow (normal-weight group) or a high-fat diet (obese group) for 6 weeks. The mice were mated, zygotes were collected from oviducts and cultured for 3 days, and six–eight-cell embryos were then selected to assess lipid content in fresh embryos and to evaluate differences in apoptosis, survival, and development rates in response to vitrification.

**Results:**

In fresh embryos from obese mice, the lipid content (0.044 vs 0.030, P<0.01) and apoptosis rate (15.1% *vs.*9.3%, P<0.05)were significantly higher, the survival rate (83.1% *vs.* 93.1%, P<0.01) on day 5 was significantly lower, and embryo development was notably delayed on days 3–5 compared with the normal-weight group. After vitrification, no significant difference was found between thawed embryos from obese and normal-weight mice in apoptosis, survival, and development rates on days 4 and 5. In both groups, pre- and post-vitrification embryo apoptosis, survival, and development rates were similar.

**Conclusions:**

This study demonstrated that differences in survival and developmental rates between embryos from obese and normal-weight mice were eliminated after vitrification. Thus, maternal obesity does not aggravate vitrification injury, but obesity alone greatly impairs pre-implantation embryo survival and development.

## Background

Obesity is an important health concern, and its prevalence has tripled in reproductive-aged women since the early 1970s. However, the impact of obesity on reproductive outcome in women using assisted reproductive technology (ART) has always been the subject of debate. Several studies of ART have associated obesity with worse reproductive outcomes than normal-weight women, including increased gonadotropin dosage [1] and cycle cancellation rate [2,3], decreased implantation and clinical pregnancy rates [3,4], increased spontaneous abortion rates [5,6], lower ongoing pregnancy and live birth rates [4,5], and increased early preterm rates [7].

Few published studies have investigated thawed embryo transfer in obese women [3]. In ART, embryo cryopreservation is a cost-effective way to increase cumulative pregnancy rates per oocyte retrieval cycle. Vitrification is a widely used and rapid method that briefly exposes embryos to high concentrations of cryoprotectant agents before ultrarapid cooling and storage in liquid nitrogen.

All embryos suffer considerable morphological and functional damage during cryopreservation, but the extent of injury and post-thaw survival and developmental rates may vary greatly among species, developmental stages, and origins. Many studies have shown that increased lipid accumulation aggravates vitrification injury in porcine and bovine embryos, and delipidation by micromanipulation or the use of chemicals can significantly reduce embryo sensitivity to chilling or cryopreservation [8-15]. The specific mechanism by which lipid accumulation in the embryo influences cryotolerance is unknown, but lipid peroxidation might account for this reduced tolerance [16]. Therefore, elevated lipid content in embryos might increase the production of free radicals and stimulate embryo death [13]. However, the lipid contents of bovine and porcine oocytes are very high (63 and 161 ng/oocyte, respectively) which can’t adequately evaluate the effects of lipid content in other species [17,18].

The lipid content of oocytes was found to be significantly higher in mice with diet-induced obesity than in a control group [19]; we can thus infer that human embryos would have greater lipid contents in obese than in normal-weight individuals, which would aggravate cryopreservation injury. Almost nothing is known about lipids in the human oocyte, except that these cells are anecdotally known to be relatively lipid poor. In this regard, human oocytes are considered to be similar to those of mice, which are estimated to have a total lipid content of 4 ng/oocyte [20].

Female mice with diet-induced obesity have shown delayed embryo development [21,22], but no available data to date have shown the impact of obesity on embryo vitrification in mice. The aim of this study was to establish a murine model of maternal diet-induced obesity and to characterize the effect of obesity on vitrification by investigating the survival rate and embryo developmental competence after thawing.

## Methods

### Mice, dietary intervention, and physiologic data collection

All experimental procedures were approved by the Ethics Committee of Sun Yat-sen University. From 5 weeks of age, female C57BL/6 mice (Laboratory Animal Services, Sun Yat-sen University, Guangzhou, China) were fed one of two diets for 6 weeks. The high-fat diet (obese group, *n* = 45) provided 45% fat, 20% protein, and 35% carbohydrate (D12451; Research Diets, Inc., New Brunswick, NJ, USA), and the matched control diet (normal-weight group; *n* = 45) provided 10% fat, 20% protein, and 70% carbohydrate (D12450B; Research Diets, Inc.). All mice were provided with food and water *ad libitum*.

To verify the establishment of the obesity model, body weight was recorded every week throughout the feeding period. Blood samples were taken at 11 weeks of age from a subset of mice (*n* = 10/group) after an 8-h fast *via* cardiac puncture under anesthesia. Blood was allowed to clot at room temperature, and samples were then centrifuged at 4000 rpm for 10 min and serum was removed. Serum samples were subjected to insulin analysis using a mouse insulin ultrasensitive enzyme-linked immunosorbent assay kit (ALPCO Diagnostics, Salem, NH, USA). Serum fasting blood glucose levels were determined using a Roche Cobas Mira automated sample system (Roche Diagnostics Corporation, Castle Hill, Australia). Cholesterol and triglyceride levels were measured using CHOD-PAP and TRG assay kits (Roche Diagnostics Corporation), respectively.

### Zygote collection and culture

All chemicals were obtained from Sigma-Aldrich Corporation (St. Louis, MO, USA) unless specified otherwise.

At 11 weeks of age, normal-weight and obese female mice were ovulated with 10 IU pregnant mare serum gonadotropin, followed 48 h later by 10 IU human chorionic gonadotropin (hCG), *via* intraperitoneal injection. The female mice were then mated with adult (10–14-week-old) male mice of the same strain and checked for the presence of a postcoital vaginal plug the following morning. The mated female mice were sacrificed by cervical dislocation 22–24 h after hCG injection, and zygotes were collected from the oviducts in HEPES-buffered α-minimal essential medium (GIBCO BRL Invitrogen Australia, Mulgrave, Australia) supplemented with 1mg/mL polyvinylpyrrolidone (PVP) to prevent sticking. Following culture in G1 media (ver. 3; Vitrolife, Göteborg, Sweden), at 66 h after hCG injection, grades I and II (even or less even blastomeres, fragmentation < 10%, intact zona pellucida) six–eight-cell embryos were selected to culture in G2 sequential medium (ver. 3; Vitrolife) to the blastocyst stag, half of the six–eight-cell embryos from each group was vitrified and thawed 5 days later, the fresh embryos served as controls.

All embryo culture media were equilibrated at 37°C in 5% CO_2_ and cultures were conducted in 30-μL drops under mineral oil.

### Embryo cryopreservation and thawing

The pretreatment, vitrification, and dilution media were based on HEPES buffer. The pretreatment solution contained 7.5% (v/v, 1.06 M) dimethyl sulfoxide (DMSO) and 7.5% (v/v, 1.3 M) ethylene glycol (EG). The vitrification solution contained 15% (v/v, 2.1 M) DMSO, 15% (v/v, 2.6 M) EG, 5.9 mg/mL Ficoll 400, and 0.58 M sucrose. The five-step dilution solutions contained 1 M, 0.5 M, 0.33 M, 0.2 M, and 0 M sucrose, respectively.

All vitrification processes were carried out at room temperature. Five to eight embryos were equilibrated in pretreatment solution for 2 min, then transferred to vitrification solution, loaded onto the tip of a 0.25-mL straw in a minimum volume of vitrification solution, and plunged into liquid nitrogen within 35–45 s.

After 5 days of storage in liquid nitrogen, the embryos were transferred to a sucrose-free medium and then subjected to a five-step thawing procedure (1 M: 1 min, 0.5 M: 1 min, 0.33 M: 2 min, 0.2 M: 3 min, 0 M: 5 min) at 37°C. After thawing, embryos were cultured in G2 medium covered with mineral oil at 37°C in saturated humidity and an atmosphere of 5% CO_2_. Embryo survival, developmental competence, and apoptosis rates were then assessed, as described below.

### Embryo developmental competence assessment

Embryo developmental competence was assessed using a phase-contrast microscope at the following timepoints after hCG injection [23]: 42 h (day 2), 66 h (day 3), 97 h (day 4), and 114 h (day 5) in the fresh group; and 31 and 48 h after embryos were thawed in the vitrified group.

Embryos were recorded when normal cell numbers and morphology were observed (i.e., two cells on day 2; six–eight cells on day 3; morula, blastocyst, or expanded, hatching or hatched blastocyst on days 4 and 5), fragmentation was <10%, and the zona pellucida was intact (except in hatching or hatched blastocysts). Embryos in which cell division events had ceased (indicating cellular arrest) were categorized as degenerate.

The zygote cleavage rate on day 2 (number of embryos cleaved on day 2/total number of one-cell embryos recovered on day 1) and six–eight-cell embryo ratio on day 3 (number of embryos scored morphologically as having six–eight cells on day 3/total number of two-cell embryos on day 2) were compared between obese and normal-weight groups. The proportions of embryos at the morula, blastocyst, and expanded, hatching and hatched blastocyst stages [number of embryos at each stage/number of six–eight-cell embryos on day 3 (fresh) or total number of thawed embryos (vitrified)] and embryo degeneration rate [number of embryos in which development had ceased/number of fresh six–eight-cell embryos on day 3 (fresh) or number of thawed embryos (vitrified)] on days 4 and 5 were compared between the fresh obese and normal-weight and vitrified obese and normal-weight groups to detect the effects of obesity and vitrification on embryo developmental potential.

### Lipid droplet staining

Lipophilic Nile Red dye was used to stain intracellular neutral lipids in the fresh six–eight-cell embryos [24]. Nile Red was reconstituted in DMSO to a concentration of 1 mg/mL and further diluted to a working concentration of 10 μg/mL in phosphate-buffered saline (PBS). To prevent embryos from sticking to pipettes, 1 mg/mL PVP was added to each solution used in this procedure. The embryos were fixed in 4% paraformaldehyde-PBS for 2 h and washed thoroughly in PBS before staining with Nile Red for 1 h at 37°C. They were then rinsed again in PBS and incubated in 1 μg/mL 4’,6-diamidino-2-phenylindole dihydrochloride (DAPI) for 15 min in the dark. After three additional washes in PBS to remove excess DAPI, embryos were mounted on 10% (w/v) poly-L-lysine–coated slides using 2-μL microdrops of Antifade (Invitrogen, Carlsbad, CA, USA).

Images of each embryo were captured using an LSM710 spectral scanning confocal microscope system (Zeiss, Göttingen, Germany). Identical magnification and gain settings were used for all samples. To examine lipid concentration, the whole volume of each embryo on the confocal image was sampled using a Z-stack consisting of a set of adjacent optical sections taken at 3-μm intervals. Total mean lipid content was calculated as the mean fluorescence intensity of all sections of each embryo using Image-Pro Plus software (ver. 6.0; Media Cybernetics, Bethesda, MD, USA).

### Assessment of DNA damage by TUNEL staining

Apoptosis rates were examined in six–eight-cell embryos before and after vitrification using terminal deoxynucleotidyl transferase-mediated 2’-deoxyuridine 5’-triphosphate-biotin nick end-labeling (TUNEL) staining [25]. The embryos were rinsed twice in 50-μL microdrops of PBS-PVP, fixed in 50-μL microdrops of 4% (w/v) paraformaldehyde in PBS for 1 h, rinsed in PBS, and then permeabilized in 0.5% (v/v) Triton X-100 for 30 min. Positive controls for the TUNEL assay were incubated in RNase-free DNase (50 U/mL) at 37°C for 1 h in the dark. Positive controls and embryos were then washed in PBS and incubated with 50 μL TUNEL reaction mixture (Roche Diagnostics Corporation, Indianapolis, IN, USA) at 37°C for 1 h in the dark. Negative controls were incubated in the absence of terminal deoxynucleotidyl transferase. After incubation, embryos were rinsed in PBS and incubated in 1 μg/mL DAPI for 15 min in the dark. After three additional washes in PBS-PVP, embryos were mounted on 10% (w/v) poly-L-lysine–coated slides using 2-μL microdrops of Antifade (Invitrogen). Images of each embryo were captured using an LSM710 spectral scanning confocal microscope system (Zeiss).

In each embryo, the total number of cells (blue fluorescence) and number of TUNEL-positive blastomeres (green fluorescence) were counted with DAPI and GFD filters, respectively, using a 40× objective. The apoptosis rate was expressed as number of TUNEL-positive blastomeres/total cell number.

### Statistical analyses

Statistical analyses were performed using the SPSS software package (ver. 13.0; SPSS Inc., Chicago, IL, USA). Normality was tested using the Kolmogorov–Smirnov test. Differences between dietary groups in body weight, serum metabolite index, and lipid droplet content were determined using the independent *t*-test. The apoptosis, survival, and development rates on each day were determined by *χ*^2^ analysis. For all analyses, *P*<0.05 was defined as statistically significant.

## Results

### Metabolic indices and lipid content in normal-weight and obese mice

**Figure 1:**
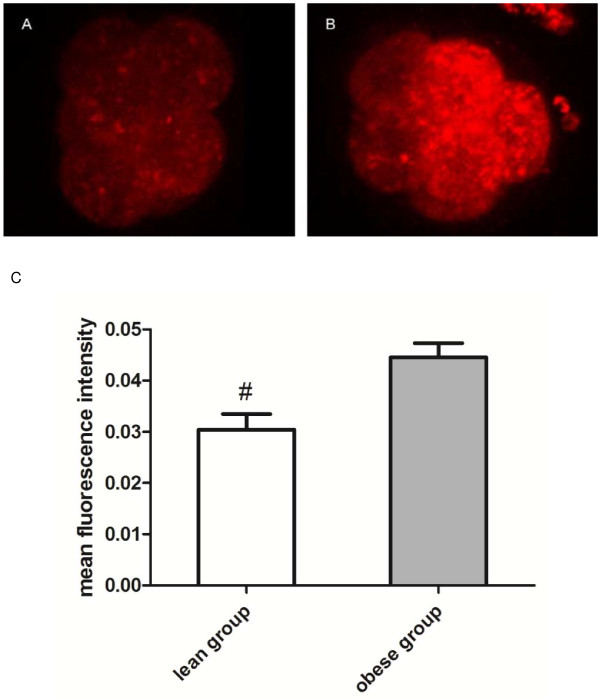
**Lipid droplet staining with Nile Red in fresh six–eight-cell mouse embryos.** Images are representative of eight-cell embryos from normal-weight (**A**) and obese (**B**) mice. (**C**) Mean fluorescence intensities of embryos (*n* = 40/group) in normal-weight and obese groups; the difference between groups was significant (*P*<0.05).

After 6 weeks, mice fed a standard diet weighed 18.9 g and those fed a high-fat diet was 21.0 g (*P*<0.001; Table 1). Compared with the normal-weight group, the obese group showed significantly higher fasting serum insulin (0.27 *vs.* 0.61 ng/mL, *P*<0.001), triglyceride (1.1 *vs.* 1.8 mmol/L, *P*<0.001), cholesterol (3.1 *vs.* 5.1 mmol/L, *P*<0.01) and glucose (4.2 *vs.* 6.0 mmol/L, *P*<0.05) levels (Table 1). The mean lipid fluorescence intensity in six–eight-cell embryos was significantly higher in the obese group than in the normal-weight group (0.044 *vs.* 0.030, *P*<0.01; Figure 1).

**Table 1:** Average body weight and metabolite concentration in normal-weight and obese female mice

	**Normal weight**	**Obese**	***P***^**1**^
Final body weight (g)	18.9 ± 1.2	21.0 ± 1.3	<0.001
Glucose (mmol/L)	4.2 ± 1.0	6.0 ± 1.5	<0.05
Insulin (ng/ml)	0.3 ± 0.8	0.6 ± 1.0	<0.001
Triglycerides (mmol/L)	1.1 ± 0.4	1.8 ± 0.5	<0.001
Cholesterol (mmol/L)	3.1 ± 1.0	5.1 ± 1.0	<0.01

*Notes:* Values are means ± standard deviation. Body weight, *n* = 45 mice/group; plasma metabolite concentrations, *n* = 10 mice per group. ^1^Independent *t*-test.

### Maternal obesity impaired pre-implantation development in fresh embryos

Zygotes recovered from obese and normal-weight female mice were cultured to the blastocyst stage to establish the effect of maternal obesity on embryo development. Cleavage to the two-cell stage was comparable between groups (*P* = 0.67; Table 2). The proportions of six–eight-cell embryos were significantly lower in the obese group than in the normal-weight group on day 3 (*P* = 0.04). This delay in development persisted on days 4 and 5 (both *P*<0.05; Table 2) of culture. On day 4 of culture, degeneration rates were similar between groups (*P*>0.05); however, the degeneration rate on day 5 was significantly higher in the obese group than in the normal-weight group (*P*<0.05; Table 2).

**Table 2:** Effect of maternal obesity and vitrification on embryo development on days 2–5 of culture

	**Fresh**		**Vitrified**		***P***^**a**^
	**Normal weight**	**Obese**	**Normal weight**	**Obese**	
No. of zygotes	30.9 ± 1.9	30.3 ± 1.3	-	-	NS
Cleavage rate (%)	64.3 ± 5.2	61.2 ± 4.5	-	-	NS
Six–eight-cell ratio (%)	75.1 ± 5.3	62.4 ± 4.2	-	-	<0.05
Morula (%)					
Day 4	19.5^b^	41.6^c^	34.2	42.2	<0.01
Day 5	5.7	9.1	10.6	10.8	NS
≥Early blastocyst (%)					
Day 4	40.2	32.5	36.8	38.5	NS
Day 5	39.1	49.4	42.1	39.8	NS
≥Expanded blastocyst (%)					
Day 4	35.7^b^	18.1^c^	25.0	16.9	<0.01
Day 5	48.3^b^	24.6^c^	36.8	32.5	<0.01
Degeneration rate (%)					
Day 4	4.6	7.8	3.9	2.4	NS
Day 5	6.9^b^	16.9^c^	10.5	16.9	<0.01

*Notes:* Data represent *n* = 10 mice/group (from 630 and 609 total zygotes in normal-weight and obese females, respectively). Cleavage rate = number of cleaved embryos on day 2/total number of one-cell embryos recovered. Six–eight-cell ratio = mean number of embryos scored morphologically as having six–eight cells/mean number of cleaved embryos on day 2. Day 4 and 5 data are expressed as mean number of embryos at each stage/mean number of six–eight-cell embryos on day 3 (fresh) or total number of thawed embryos (vitrified). Degeneration rate = number of embryos that have ceased development/number of six–eight-cell embryos on day 3 (fresh) or number of thawed embryos (vitrified). ≥Early blastocyst = early-, mid-, or late-stage blastocyst. ≥Expanded blastocyst = expanded, hatching, or hatched blastocyst. NS = not significant; ^a^Chi-squared test; ^b^*vs.*^c^, *P*<0.01.

### Pre- and post-vitrification degeneration and development in six–eight-cell embryos

The degeneration rates and developmental potentials of fresh and thawed embryos were comparable in the obese and normal-weight groups on days 4 and 5 of culture (Table 2). Vitrification had little effect on embryo survival and developmental competence in both diet groups.

### Developmental differences between embryos from obese and normal-weight mice were eliminated after vitrification

Degeneration rates and developmental competence were similar in the obese and normal-weight groups on days 4 and 5 after the embryos were thawed (Table 2), in contrast to the developmental difference observed in fresh embryos. These results demonstrate that differences in embryo survival and development between obese and normal-weight groups were eliminated after vitrification.

### Pre and post-vitrification apoptosis rates in six–eight-cell embryos

Before freezing, the apoptosis rate in six–eight-cell embryos was significantly higher in the obese group then in the normal-weight group (15.1% *vs.* 9.3%, P<0.05). After freezing, apoptosis rates were more similar between groups (18.3% *vs.*13.0%, P>0.05). Pre- and post-vitrification rates did not differ significantly in either group.

**Figure 2:**
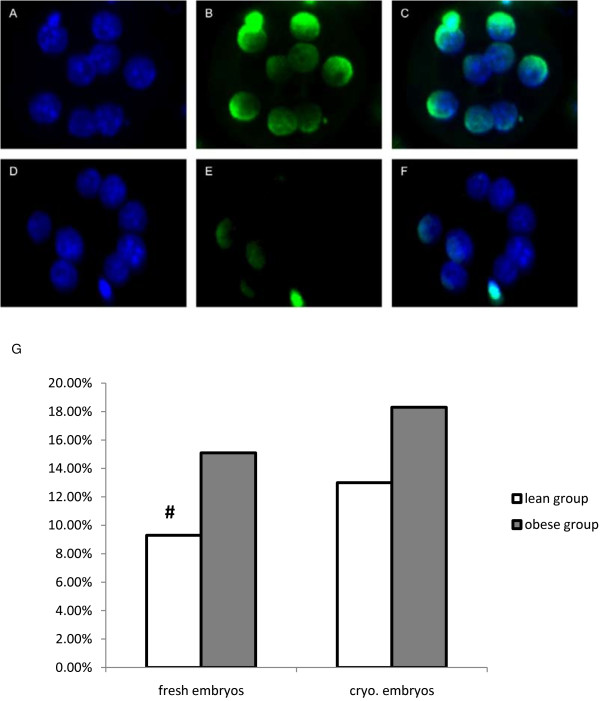
**Representative confocal images illustrating apoptosis frequency in six–eight-cell mouse embryos subjected to TUNEL analysis.** Positive control embryo nuclei in the blue (**A**) and green (**B**) fluorescence channels, and (**C**) merged image. Embryo nuclei in the blue (**D**) and green (**E**) fluorescence channels, and (**F**) merged image. (**G**) Apoptosis rates of fresh and thawed embryos in the normal-weight and obese groups. Apoptosis rate = TUNEL + blastomeres/total number of blastomeres. Each group contained 40–50 embryos. The apoptosis rate was significantly higher in fresh embryos in the obese (*vs.* normal-weight) group, but did not differ between groups of thawed embryos.

Vitrification had no effect on embryo apoptosis. The elevated lipid content in six–eight-cell embryos from obese mice did not aggravate cryogenic injury, but obesity alone increased apoptosis rates in fresh embryos (Figure 2).

## Discussion

This study documented cryotolerance in six–eight-cell embryos in a murine model of diet-induced obesity. High lipid content in embryos from obese mice did not aggravate cryodamage during vitrification, although obesity alone impaired embryo survival and development. Differences in the survival and development of embryos from obese and normal-weight mice were eliminated after vitrification.

In this study, fasted obese mice showed significantly elevated cholesterol, triglyceride, and blood glucose levels, as well as hyperinsulinemia, compared with normal-weight mice. This abnormal metabolic environment may have impaired early embryo development. Minge *et al.*[21] found that embryos from obese mice had significantly reduced on-time progression compared with those from control mice, and that altered granulosa cell phenotype and ovarian dysfunction were caused by compromised metabolism due to excessive caloric intake in mice with diet-induced obesity; these changes were also observed under conditions of genetic mutation–induced obesity [26-29]. The underlying mechanism may be inhibited expression of lanosterol 14 alpha-demethylase caused by a high-fat diet [30]; the lanosterol 14 alpha-demethylase–mediated sterol pathway is involved in ovarian development [31], quality control of oocyte meiotic maturation [32-34], fertilization, and embryo developmental competence [35,36]. Another possible mechanism may be low levels of follicle-stimulating hormone (FSH) and luteinizing hormone (LH) in obese mice. Inappropriately low LH and FSH concentrations are prevalent in patients with obesity and other features of metabolic syndrome (hypertension and hyperlipidemia), this prevalence may be the result of insulin resistance at the level of the gonadotropin-releasing hormone (GnRH)-secreting neuron because insulin facilitates the secretion of GnRH from neuronal cell cultures [37]. LH and FSH are two important stimulators of eggs development and maturation [38,39].

The compromised metabolic environment induced by a high-fat diet significantly increased the lipid contents of mouse oocytes [19] and embryos, as our research confirmed. A high-fat diet significantly reduced mitochondrial membrane potential in mature oocytes [19], with significant consequences for developmental competence [40-43]. Cumulus-oocyte complexes (COCs) from mice fed a high-fat diet showed increased expression of the endoplasmic reticulum (ER) stress marker genes ATF4 and GRP78, and increased apoptosis rates were observed in granulosa and cumulus cells of mice fed a high-fat diet [19]. Thus, lipid accumulation, ER stress, mitochondrial dysfunction, and apoptosis are markedly increased in COCs from mice fed a high-fat diet. These results indicate that lipotoxicity may also occur in the ovarian cells and oocytes of obese mice, which may contribute to the reduced survival and delayed development observed in response to obesity.

Events that exert any kind of stress on an embryo, such as thermal stress [44,45], oxidative stress [46], unfavorable conditions in culture media composition [47,48], and cryopreservation, are potential causes of apoptosis. Mammalian oocytes and embryos have shown high survival rates after vitrification [49,50], in which ultrarapid cooling produces ice crystal–free, solid, glasslike structures. The high survival rate after thawing and the large proportion of normal spindle/chromosome configurations [49,50] suggest that vitrification does not adversely affect human embryo development, including the ability to form spindles and continue normal cell division. In the present study, high lipid content in embryos from obese mice significantly impaired embryo developmental potential, but the stress generated by vitrification had no effect on embryo survival or developmental competence in the obese or normal-weight group. Thus, obesity, but not vitrification, negatively affected embryo development and survival.

This article reports the first published research on the effect of lipids on vitrification in a murine model. In contrast to studies conducted in other species, we found no difference in vitrification injury associated with dietary-induced obesity. Discrepancies in findings between studies in mice and domestic animal species may be explained by the markedly high lipid content in embryos from the latter; the total lipid content per oocyte is 4 ng in mice [20], in contrast to 161 ng in pigs, 63 ng in cattle, and 89 ng in sheep [17,18]. Thus, lipid toxicity induced by high organ lipid content may be less pronounced in mouse embryos than in domestic animal embryos.

In contrast to the differences observed in fresh embryos, survival and developmental potential were similar in thawed embryos from obese and normal-weight mice. These results are in accordance with patterns observed in human ART outcome: the odds of pregnancy failure increase with body mass index (BMI) among women with autologous fresh cycles (BMI ≥ 25.0 kg/m^2^, OR = 1.10/cycle; BMI ≥ 50.0 kg/m^2^, OR = 2.29/cycle) but not among those BMI < 45 kg/m^2^ with autologous thawed cycles; however significant difference from BMI > 45 kg/m^2^ (BMI = 45.0–50.0 kg/m^2^, OR =1.45/cycle) [3]. Taken together, these results suggest that embryonic and endometrial factors may play a role in the poor ART outcomes associated with obesity in autologous fresh cycles, whereas endometrial factors may play a major role in autologous thawed cycles because embryo quality differences are eliminated after vitrification.

## Conclusions

Obesity had no effect on embryo vitrification injury in a murine model, but it greatly reduced embryo development and impaired embryo survival. These differences were eliminated after vitrification. The study renews our knowledge about the effect of high lipid content on vitrification injury. Future research should seek to identify differences in organelle function, total cell numbers, and blastocyst implantation potential after the vitrification of six–eight-cell embryos to elucidate the conclusion more deeply.

## Competing interests

The authors declare that they have no competing interests.

## Authors' contributions

XYL conceived this study. WHM and XY designed the protocols, carried out all experiments, and analyzed the data. WHM drafted the manuscript. All authors read and approved the final manuscript.
